# Editorial: Insights in virus and host: 2021

**DOI:** 10.3389/fcimb.2023.1190338

**Published:** 2023-04-11

**Authors:** John Hiscott, Curtis R. Brandt

**Affiliations:** ^1^ Laboratorio Pasteur, Istituto Pasteur-Fondazione Cenci Bolognetti, Roma, Italy; ^2^ Department of Ophthalmology and Visual Sciences, University of Wisconsin School of Medicine and Public Health, Madison, WI, United States; ^3^ Department of Medical Microbiology and Immunology, University of Wisconsin School of Medicine and Public Health, Madison, WI, United States

**Keywords:** KHSV, RIO kinase, Junin virus, Dengue virus, Chikungunya virus, Porcine epidemic diarrhea virus

We have entered the third decade of the 21st Century with a unique perspective – a post-pandemic world in which the exceptional scientific achievements of the last three years have brought the COVID-19 pandemic under control. This increased awareness extends to the general population where knowledge of virus-host interactions now incudes an understanding virus biology and pathogenesis, as well as the impact of SARS-CoV2 mutational alterations on infectivity and vaccine efficacy. The immense global effort in molecular virology, cellular immunology, vaccine development and antiviral drug design have highlighted the continuing need to understand better the intricate mechanisms controlling virus-host interactions and their pathogenic consequences. In addition, technological developments including single cell OMICs to dissect the immune response, cryo-EM to elucidate structure-function relationships, and AI-based programs to understand protein structure and folding have directed numerous advances in the study of Virus-Host relationships. In this Special Topic, Frontiers asked our Associate Editorial Board members to highlight the latest advancements and perspectives in research across the field, with primary research articles, editorials, or reviews. In this special topic we present 5 primary research articles involving studies utilizing multiple different viruses and investigating multiple aspects of viral infection.

The first article by Long et al. focuses on Atlastin proteins (ATLs), large dynamin-related GTPases that regulate ER membrane function, and their role in promoting KSHV lytic activation and infection. Overexpression of ATLs enhances lytic infection while deletion has the opposite effect. This response is also correlated with induction of ER stress and that ER stress has positive effects on viral infection. Furthermore, knockdown of CHOP and BIP, two proteins involved in stress responses, countered the promoting effect of ATLs, further supporting a role for ER stress in modulating virus infection. This study adds to the growing body of work on the role of the ER and ER stress responses in viral infection.

White et al. focuses on the role of RIO kinase splicing in Rift Valley Fever Virus and the connection between alternative splicing of RIO and innate resistance to viral infection. During infection, RIOK3 mRNA is alternatively spliced, producing an isoform that interferes with IFN-β signaling. They show that the host cell splicing factor TRA2-β is a critical regulator of spliced RIOK isoforms. TRA2-β interacts with RIOK pre-mRNA to regulate constitutive splicing while a lack of TRA2-β led to increased alternative splicing. Interestingly, TRA2-β was also alternatively spliced in infected cells, reducing the amount of TRA2-β protein. These results point to regulation of splicing as a mechanism to promote immune evasion by the virus and may also serve as a mechanism whereby the host can dampen potentially overenthusiastic immune responses.

The third contribution by Fitzpatrick et al. examines Junin virus (JUNV), a hemorrhagic fever virus that is an important pathogen in South America. The authors use reverse phase Protein Microarrays to compare global alterations of the host proteome between an attenuated vaccine strain and a wild type strain of JUNV. Examination of proteome differences at various time points post-infection revealed that 14 proteins were significantly altered by infection. Several proteins were post-translationally modified by phosphorylation including MAPK, HSP27, and NF-κB. Activation of cellular kinases could be expected during entry as this is commonly found with several viruses that activate NF-κB. The modification of HSP27 suggests that the chaperone function, or perhaps other functions of HSP27, may be involved early in infection. They also showed that knockdown of p38MAPK, using a specific inhibitor, or siRNA-mediated knockdown of HSP27 decreased JUNV replication and that phosphorylation of HSP27 by p38MAPK may play a role in events early in infection.

In tropical and sub-tropical regions of the world, both Dengue and Chikungunya viruses co-circulate, leading to the potential for co-infection. The study of co-infections with two different viruses remains an underexplored area of research where infection with one virus may inhibit infection by a second virus either concomitantly or sequentially, referred to as viral interference. Taraphdar et al. present studies of co-infection of cultured liver cells with Dengue and Chikungunya using various scenarios of co-infection or sequential infection. If DENV was present prior to, or upon superinfection, CHIKV replication was enhanced. Co-infection with both viruses reduced infection but DENV replication was more significantly reduced. They also found that low levels of antibody enhanced infection with both viruses. Overall, CHIKV had a fitness advantage compared to DENV in liver cells and prior infection with DENV may enhance CHIKV disease severity.

In the final paper of this Research Topic, Ren et al., investigate the antiviral effect of the natural product cinchonine on Porcine Epidemic Diarrhea virus. Cinchinone treatment resulted in a significant reduction in viral replication that appeared to manifest at an early stage in infection. Cinchinone also induced autophagy in cells. Furthermore, the antiviral effect was reduced in the presence of the autophagy inhibitor 3MA indicating that cinchonine acts at least partially by targeting autophagy. This article further supports the possibility that autophagy may be a potentially valuable antiviral target.

In summary, this diverse collection of primary research papers advances our understanding of the role of cellular processes in viral infections and expands our understanding of host-virus interface. The long-term goal of these research endeavors is to lay the foundation for the development of novel antiviral strategies to control viral infection and pathogenesis. With the hindsight of the COVID-19 pandemic, we now clearly recognize the need for these advances.

## Author contributions

CB prepared the initial draft that was edited by JH. All authors contributed to the article and approved the submitted version.

